# Childhood Asthma Utilization Rates in a Nonsmoking Population of Utah Compared to State and National Rates

**DOI:** 10.5402/2011/750213

**Published:** 2011-10-12

**Authors:** Lisa H. Gren, Brooke Taylor, Joseph L. Lyon

**Affiliations:** Department of Family and Preventive Medicine, The University of Utah, Salt Lake City, UT 84108, USA

## Abstract

Risk factors, such as parental smoking, are commonly associated with increased asthma symptoms and hospitalizations of children. Deseret Mutual Benefits Administrators (DMBA) is the health insurer for employees of The Church of Jesus Christ of Latter-day Saints and their families. Due to religious proscription, employees abstain from alcohol and tobacco use, creating a cohort of children not exposed to parental smoking. Calculation of hospitalization rates for DMBA, Utah, and the US were made in children to compare rates between a nonsmoking population and general populations. Compared to DMBA, rate ratios for asthma hospitalization and emergency department asthma visits were higher for the US and Utah. The incidence of hospital outpatient department and physician office visits was significantly greater for the US population compared to the DMBA. This study demonstrates a decreased need for health services used by children not exposed to second-hand smoke.

## 1. Introduction

Asthma is a chronic respiratory condition that affects over 7 million United States (US) children [1]. Acute episodes are characterized by airway constriction and excess production of mucus and may require medication to restore normal breathing. Several different triggers have been found to set off episodes, including cold or dry air, dust, pollen, pollution, physical activity, stress, and cigarette smoke. 

Parental cigarette smoking influences children's asthma-related symptoms. Children are more vulnerable to tobacco smoke than adults because their respiratory and immune systems are not completely developed [2]. As early as 1992, the Environmental Protection Agency reported that environmental tobacco smoke (ETS) is causally associated with 1 million episodes and increased severity of symptoms among asthmatic children [3]. In addition, ETS is a risk factor for new asthma cases in children [4]. In the US in 1997, figures for childhood illness and death attributed parental smoking with an estimated excess of 1.8 million outpatient visits for asthma and an excess of 14 asthma deaths [5]. A worldwide study conducted in 2004 found that 603,000 deaths were attributable to ETS exposure, 28% of which were in children [6]. 

Deseret Mutual Benefits Administrators (DMBA) is the health insurer for employees of The Church of Jesus Christ of Latter-Day Saints (LDS) and their families, with electronic data for 62,000 individuals. Due to religious proscription, those who work for the LDS church are required to abstain from alcohol and tobacco use, creating a cohort of children not exposed to parental smoking. Utah's rate for lung cancer traditionally is about half that of the US [7]. Non-LDS in Utah have 1.6x (male) and 2.2x (female) higher rates of smoking-related cancers, compared to their LDS counterparts [8]. Not everyone who is listed on the membership records of the LDS church adheres to the teachings regarding abstinence from smoking; however, those employed by the church, and therefore under DMBA coverage, are required to state they follow the nonsmoking requirement. Thus, we would expect to see a gradient of smoking-related disease, with the lowest rates found in the DMBA population and the highest rates in the US. The rates for Utah, with its large LDS population, should be intermediate between the DMBA and US rates. This study compares the childhood asthma hospitalization rates of a nonsmoking population (DMBA enrollees) to Utah and US hospitalization rates to determine the impact of smoking on these rates. It also examines the effect of smoking on utilization of asthma-related outpatient medical services, including emergency department (ED) visits.

## 2. Methods

### 2.1. Study Population

The DMBA insurance claims database was used to estimate childhood hospitalization rates from 1997–2002 among a nonsmoking population. The DMBA was established in 1970 to provide insurance to the employees of the LDS Church and their families. Electronic data recording began in 1995 and was completed by the end of 1996. Study data collection covers the years 1997–2002. The DMBA is a national insurance claims database that insures approximately 62,000 members a year with nearly equal numbers of males and females. Utah residents under the age of eighteen comprise approximately 35% of the database, and analysis was limited to this population. The DMBA population has little employment turnover, estimated at less than 5% per year, providing a stable population for analyses. The majority of turnover occurs because of the addition of newborns, the loss of young adults who marry, move out of the parental home, or reach age 26, and individuals who become eligible for Medicare. The University of Utah's Institutional Review Board approved this study as low risk. 

### 2.2. National Hospital Discharge Survey

The National Hospital Discharge Survey (NHDS) is an annual national probability survey that collects information on characteristics of inpatients discharged from non-Federal short-stay hospitals in the United States. The NHDS collects data from a sample of approximately 270,000 inpatient records acquired from about 500 hospitals. Reported data include number and rate of discharge and average length of stay by age, sex, and geographic region. Only first listed diagnoses of International Classification of Disease, 9th Revision, Clinical Modification (ICD-9), are used to define disease [9]. Data for this study covers the years 1997–2001.

### 2.3. National Ambulatory Medical Care Survey

The National Ambulatory Medical Care Survey is an annual national survey that is designed to collect data on the use of ambulatory care services in the United States. Results are based on a national sample of visits to nonfederally employed office-based physicians who are primarily engaged in direct patient care. Specially trained interviewers visit the physicians prior to participation to train them in data collection procedures. Each physician is randomly assigned a 1-week reporting period. Data include information about physician diagnoses, medications ordered or provided, patient demographics, and services provided [10]. Data for this study covers the years 1997–2002.

### 2.4. National Hospital Ambulatory Medical Care Survey

The National Hospital Ambulatory Medical Care Survey is a survey designed to collect data on the utilization of ambulatory services in hospital emergency and outpatient departments. Findings are based on a national sample of visits to emergency and outpatient departments of noninstitutional and short-stay hospitals, exclusive of federal hospitals, military hospitals, Veterans Administration hospitals, psychiatric institutions, and long-term care facilities. Specially trained interviewers visit hospitals prior to participation to explain survey completion procedures. Hospital staff complete annual surveys during a 4-week period. Collected data include patient demographics, physician diagnoses, services and procedures provided, and medication provided [11]. Data for this study covers the years 1997–2002.

### 2.5. National Health Interview Survey

The National Health Interview Survey (NHIS) is an annual survey conducted by the Centers for Disease Control and Prevention's National Center for Health Statistics and provides national estimates for a broad range of health measures for children less than 18 years of age. Each year, a representative number of households across the United States are selected using a multistage cluster sample design. The annual NHIS questionnaire is administered to the entire household. Child health information, utilization of health care services, household composition, and sociodemographic characteristics are ascertained by asking a knowledgeable family member, aged 18 years or older, who resides in the household. Two questions are asked to determine the prevalence and frequency of asthma: “ever told had asthma,” and “had an asthma attack in the last 12 months” [12]. Data for this study covers the years 1997–2002.

### 2.6. Utah's Indicator-Based Information System for Public Health

The Utah Indicator-Based Information System for Public Health contains data on all hospitalizations from the 49 Utah hospitals operating during the study period. Billing, medical, and demographic information describing a patient, the services received, and inpatient charges are collected quarterly [13]. Hospital discharge diagnosis, based on ICD-9 codes was used to identify inpatient hospitalizations for asthma. Data for this study covers the years 1999–2002.

### 2.7. Asthma Case Definition

Asthma cases were defined using the primary ICD-9 diagnosis codes 493.00–493.99. DMBA asthma services prevalence was determined by dividing all individuals with an asthma claim within a calendar year by the population of those <18 in that year. Multiple claims could be generated at a single visit in order to provide for differing points of service. In our data, these claims were associated and counted as a single visit. However, if treatment for asthma was sought more than once during a year, each visit was counted separately. Stratifications were made by sex, age (<5, 5–11, 12–17 years), calendar year, and season (Mar–May, Jun–Aug, Sept–Nov, Dec–Feb). Ambulatory visits were broken down into physician office visits, outpatient visits, and ED visits in order to establish utilization rates. Outpatient and physician office visit information was not available for Utah.

### 2.8. Statistical Analysis

Utilization rates in DMBA were compared to the state and national data sources by calculating rate ratios (RR) with 95% confidence intervals (CI) [14]. One-way analysis of variance (ANOVA) was conducted using SAS, version 9.1, to determine differences in age-specific seasonal asthma cases. The level of significance was set at alpha = 0.05 using a two-tailed test.

## 3. Results

Baseline characteristics for the DMBA population are shown in Table 1. The average number of health care utilizations for asthma in children was 2.03 per year (95% CI 1.74–2.34). Age- and sex-specific hospital discharge rates for asthma are presented in Table 2 for the DMBA and comparison populations. A similar trend was seen in all populations, with asthma discharge rates for males 30–50% higher than for females. The DMBA had the lowest discharge rate for all ages across both sexes, with Utah rates intermediate between DMBA and US rates. Hospitalizations among US children were 5 times higher (95% CI 3.65–6.84) than among DMBA children. Hospitalizations among Utah children were 3.34 times higher (95% CI 2.44–4.47) than among DMBA children. Average length of stay was similar between the DMBA and comparison populations, at about three days (data not shown). 

 A similar pattern was seen for visits to ED and outpatient clinics (Table 3). Relative to children in DMBA, emergency department visits were 4.00 (95% CI: 3.50–4.56) times higher for US children and 1.20 (95% CI: 1.05–1.37) times higher for Utah children. Relative to children in DMBA, hospital outpatient department visits were 5.56 (95% CI: 4.64–6.65) times higher for US children. Relative to children in DMBA, physician's office visits were 1.65 (95% CI: 1.60–1.71) times higher for US children. Data were not available to make Utah to DMBA comparison for hospital outpatient or physician's office visits. 

**Figure 1:**
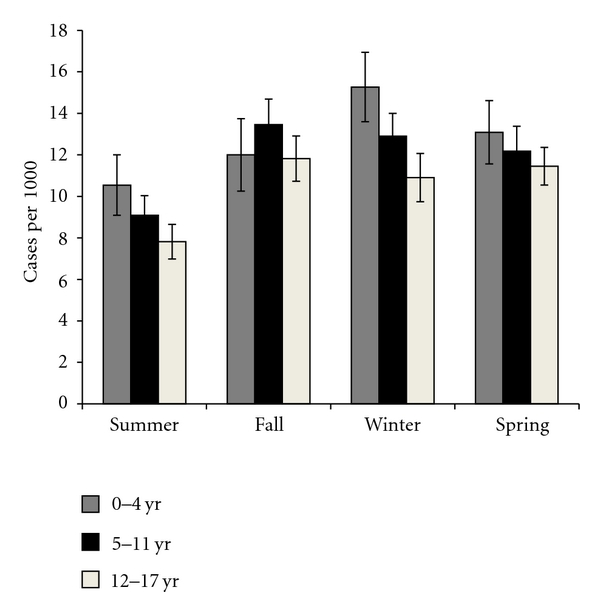


As seen in Figure 1, utilization of health services for asthma was generally highest for those aged 0–4 years, and there was no significant variation in utilization by season in this age group (*P* = 0.19). There was seasonal variation and utilization for older age groups, with summer having the lowest rates for both ages 5–11 (*P* = 0.005) and 12–17 (*P* = 0.05). 

The prevalence of asthma was estimated for DMBA as the proportion of children with any claim for asthma-related services (ICD-9 493.00–493.99), and compared with NHIS survey prevalence of asthma for the US (Table 4). The prevalence for DMBA children was relatively constant across the age groups 0–4 years, 5–11 years, and 12–17 years, while there was a noticeable increase in prevalence for US children as age increased. The relative prevalence of asthma was 3–6 times higher for US children compared to DMBA children.

## 4. Discussion

### 4.1. Study Summary

All measures of asthma service utilization were lower in the DMBA pediatric population than in comparison groups in the US and Utah. We hypothesized that asthma services utilization would follow the same trend seen for other smoking-associated diseases, with DMBA having the lowest rate, Utah having an intermediate rate, and the US having the highest rate, based on the prevalence of smoking in these populations. This trend was documented for asthma hospitalizations (3.3-fold and 5.0-fold higher in Utah and the US, resp., compared to DMBA) and ED visits (20% higher in Utah and 4-fold higher in the US). For outpatient visits, data were only available for the DMBA and US populations. Visits to hospital outpatient departments were 5.6-fold higher for US relative to DMBA children, and visits to physician's offices were 1.7-fold higher for US relative to DMBA children. The prevalence ratio for asthma services used, regardless of location of care, was higher for US children than for DMBA children, ranging from 3.1 for children age 0–4 years to 6.4 for children age 12–17 years. 

One of this study's strengths is that it included males and females in a very stable population, ranging from 0–17 years of age. Those covered under the DMBA insurance have a low turnover rate, estimated at less than 5% per year, providing a stable population for analyses. The DMBA population was limited to the insured employees and their families, who work for and are members of the Church of Jesus Christ of Latter-Day Saints. Members of this religion abstain from alcohol and tobacco use, creating a cohort of children not exposed to parental smoking. This created a unique opportunity to use population-based data to study the impact of environmental tobacco smoke on childhood asthma hospitalization and health care service utilization.

### 4.2. Potential for Biases

While there is no direct information available on smoking for individuals who are covered by the DMBA, it is a requirement to abstain from smoking to retain employment. The low lung cancer rates in the DMBA population support the premise that it is primarily composed of nonsmokers. Additionally, studies have documented rates of smoking-related cancers in LDS populations that are about half that seen in the US and about two-thirds that seen in Utah [8, 15]. The possibility exists that despite the employed parent being required to abstain from tobacco use, a spouse or child could potentially be a smoker and still be covered by the DMBA. This would reduce the exposure difference between the DMBA and comparison groups, leading to an underestimate of the RR for children living in nonsmoking households and would not change our overall conclusion. 

The DMBA is an insured population, so the use of services could potentially be higher than the general population where not all individuals have insurance coverage. The net effect of this bias would be that the RRs comparing other populations to DMBA are an underestimate of the actual amount of services used in the comparison populations. Thus, this would not alter our conclusion. 

Employed populations often have improved health when compared to the general population, specifically because they are healthy enough to be employed. We believe that the health benefits of employment are unlikely to explain the lower childhood asthma rates, as the children themselves are, for the most part, not employed. Higher socioeconomic status within the DMBA population also does not explain their lower childhood asthma rates, since the majority (70.6%) of the employed individuals' income is $60,000 or less per year. 

This study shares the limitations of all nonrandomized, observational studies, including the possibility of selection bias and unadjusted confounding. Using an insurance claims database is more accurate than voluntary self-reporting of illness, since physicians rely on ICD-9-CM codes for reimbursement. Diagnostic discharge data were used in this study in order to reflect actual diagnosis rather than claims attempting to rule out asthma, which could have overestimated the use of services. While discharge data are subject to diagnostic inaccuracy, these errors are likely to be distributed randomly through all databases and should not bias our comparisons. Comparisons made between the DMBA, the state of Utah, and the US were based on standard definitions of disease, and there should be no bias associated with the comparisons. Comparisons in this study were made between age- and sex-specific groups to minimize confounding due to these factors.

### 4.3. Comparison with Previous Studies

Akinbami and Schoendorf studied national asthma health care utilization in physician offices, hospital EDs, and hospital outpatient departments. Their reported asthma rates for hospitalization, ED and outpatient visits, and office visits were similar to the US rates we have reported [16]. DMBA rates were significantly lower than those reported by Akinbami, as well as Utah, and US rates (Table 3). 

In a 2004 study conducted by Sturm et al., after adjusting for sex, race, socioeconomic status, and a variety of environmental factors, those who were exposed daily to ETS were 1.72 times more likely to have active, diagnosed asthma (defined as self-reported asthma symptoms in children with a previous diagnosis of asthma) and 1.86 times more likely to have experienced wheezing in the last 12 months. The risk of asthma diagnosis and increased asthma symptoms associated with ETS illustrates a dose response pattern, with a small but significantly increased risk among those exposed less than once a month and a much larger risk among those exposed daily [4].

Several studies have reported on the seasonal variation in asthma. Fleming found that rates for hospital admissions and asthma episodes were highest during September and lowest during August for children in both age groups 0–4 and 5–14 [17]. Weiss reported that hospitalizations peaked between September and November for those age 5–34 years [18]. Khot and Burn also found that admissions peaked in the fall [19]. Our study found that rates in the DMBA for children 5–11 and 12–17 peaked in the fall (September to November), but that rates for the age group 0–4 were highest in the winter months (November to February). The difference between our study and that of Khot and Burn for the age group 0–4 might be attributed to regional differences between Utah and Great Britain. In Utah, children without fully developed immune and respiratory systems are likely to be more at risk due to the harsh winter climate. This could lead to a greater frequency of upper respiratory infections and asthma prevalence in the winter for the youngest age group. 

Several studies have sought to address the effects of parental smoking and medical visits for childhood asthma. Thyrian et al., Evans et al., and Jindal and Gupta studied the impact of ETS on children and found that smoking in the home was significantly associated with an increase of ED visits and hospitalizations [20–22]. Mackay et al. studied the influence of smoke-free legislation on admissions for childhood asthma. Before legislation was implemented, they found admissions increasing at 5.2% per year; however, after legislation implementation in March 2006, a decrease of admissions of 18.2% per year was found relative to prelegislation rates [23]. Wilson et al. conducted a randomized controlled trial highlighting a reduction in acute asthma medical visits and overall healthcare utilization for children whose parents were assigned to smoking-cessation classes [24]. Similar to our study, these studies document that reductions in ETS exposure reduce outpatient visits, ED visits, and hospitalizations in children.

## 5. Conclusion

Utilization rates for hospitalization, ED, outpatient department, and physician office visits were significantly lower in the nonsmoking group formed by the DMBA population when compared to the US Utilization rates for hospitalization and ED visits for Utah children were intermediate between DMBA and US rates. The comparison of the DMBA to state and national rates demonstrates the magnitude of the difference between populations exposed to ETS versus those who are not exposed.

## Figures and Tables

**Table 1:** Characteristics for the DMBA population from 1997–2002 for individuals less than 18 years of age that reside in Utah.

	1997	1998	1999	2000	2001	2002	Combined
DMBA population	13,318	13,511	14,128	14,187	14,470	14,460	84,074

Asthma cases							
0–4 years	46	56	63	60	81	95	401
5–11 years	104	111	124	114	124	135	612
12–17 years	131	142	148	117	163	155	856
Total	281	309	335	291	368	385	1,969

Mean age							
All children	10.30	10.21	10.00	9.80	9.72	9.62	9.94
Asthma cases	10.23	10.39	10.05	9.77	9.79	9.46	9.95

Mean asthma Utilizations^a^ per individual	2.16	2.10	1.96	1.93	2.14	1.90	2.03

**^a^**Any visit to a hospital, emergency department, outpatient hospital department, or a physician office.

**Table 2:** Age- and sex-specific asthma hospital discharge^a^ rates and rate ratios for DMBA, Utah, and United States, 1997–2001.

		Discharge rate per 1000			Rate ratio^b^ (95% confidence interval)	
	Age (yr)	DMBA	Utah	US	Utah	US
Male	0–4	2.08	4.36	7.06	2.10 (1.24–3.54)	3.39 (2.01–5.73)
	5–11	0.70	1.34	2.74	1.91 (0.99–3.69)	3.91 (2.04–7.52)
	12–17	0	0.98	1.04	—^c^	—^c^
	All ages	0.65	2.11	3.35	3.25 (2.16–4.89)	5.15 (3.43–7.75)

Female	0–4	1.08	2.73	4.16	2.53 (1.20–5.31)	3.85 (1.84–8.08)
	5–11	0.65	0.95	1.82	1.46 (0.73–2.93)	2.80 (1.40–5.60)
	12–17	0.07	1.34	1.14	19.14 (2.88–127.10)	16.29 (2.46–108.00)
	All ages	0.47	1.6	2.22	3.40 (2.20–5.27)	4.72 (2.90–7.71)

Total	0–4	1.59	3.57	5.61	2.25 (1.46–3.45)	3.53 (2.30–5.41)
	5–11	0.68	1.15	2.28	1.69 (1.05–2.72)	3.35 (2.09–5.38)
	12–17	0.03	1.15	1.09	38.33 (5.60–290.33)	36.33 (4.80–275.00)
	All ages	0.56	1.87	2.80	3.34 (2.44–4.47)	5.00 (3.65–6.84)

^a^ICD-9 codes 493.00–493.99 as reported by the Utah Indicator-Based Information System for Public Health (Utah rates) and the National Hospital Ambulatory Medical Care Survey (US rates)

^b^Referent category is DMBA

^c^No cases were reported in DMBA, precluding comparison to DMBA.

**Table 3:** Average annual age-specific asthma^a^ utilization rates for DMBA, Utah, and United States by location of care, 1997–2002.

		Utilization rate per 1000			Rate ratio (95% confidence interval)	
Location	Age (yr)	DMBA	Utah	US	Utah	US
Emergency department^b^	0–4	4.68	4.56	15.70	0.97 (0.78–1.22)	3.35 (2.68–4.19)
	5–11	2.46	2.61	9.76	1.07 (0.85–1.34)	3.97 (3.16–4.97)
	12–17	1.71	2.19	6.82	1.28 (1.00–1.64)	4.00 (3.12–5.09)
	All ages	2.56	3.07	10.39	1.20 (1.05–1.37)	4.00 (3.50–4.56)

Hospital outpatient department^b^	0–4	1.23	—^d^	10.69	—^d^	8.69 (5.61–13.44)
	5–11	1.35	—^d^	7.79	—^d^	5.77 (4.25–7.82)
	12–17	1.53	—^d^	5.40	—^d^	3.53 (2.72–4.57)
	All ages	1.40	—^d^	7.78	—^d^	5.56 (4.64–6.65)

Physician office^c^	0–4	33.73	—^d^	70.04	—^d^	2.08 (1.91–2.25)
	5–11	34.12	—^d^	60.45	—^d^	1.77 (1.67–1.88)
	12–17	35.94	—^d^	38.61	—^d^	1.07 (1.02–1.13)
	All ages	34.85	—^d^	57.67	—^d^	1.65 (1.60–1.71)

^a^ICD-9 codes 493.00–493.99

^b^National Hospital Ambulatory Medical Care Survey

^c^National Ambulatory Medical Care Survey

^d^Data not available for Utah.

**Table 4:** Age-specific asthma services prevalence per 1000 and rate ratios for the DMBA^a^ and United States^b^, 1997–2002.

	Prevalence per 1000		
Age (yr)	DMBA	US	Rate ratio (95% confidence interval)
0–4	24.73	75.79	3.06 (2.77–3.37)
5–11	23.36	126.15	5.40 (5.02–5.80)
12–17	22.92	146.23	6.38 (5.97–6.82)

^a^DMBA prevalence was determined by dividing all individuals with an asthma claim (ICD-9 codes 493.00–493.99) by the population of those less than18 years of age in that year.

^b^National Health Interview Survey.
